# IL-8/CXCL8 Upregulates 12-Lipoxygenase Expression in Vascular Smooth Muscle Cells from Spontaneously Hypertensive Rats

**DOI:** 10.4110/in.2009.9.3.106

**Published:** 2009-06-30

**Authors:** Jung Hae Kim, Young Jin Kang, Hee Sun Kim

**Affiliations:** 1 Department of Microbiology, College of Medicine, Yeungnam University, Daegu, Korea.; 2 Department of Parmacology and Aging-associated Vascular Disease Research Center, College of Medicine, Yeungnam University, Daegu, Korea.

**Keywords:** IL-8/CXCL8, 12-lipoxygenase, rat vascular smooth muscle cell

## Abstract

**Background:**

We previously demonstrated remarkable differences in the expression of IL-8/CXCL8 in aortic tissues and vascular smooth muscle cells (VSMC) from spontaneously hypertensive rats (SHR) compared to VSMC from normotensive Wistar-Kyoto rats (WKY). In the present study, we investigated the direct effect of IL-8/CXCL8 on expression of 12-lipoxygenase (LO), a hypertensive modulator, in SHR VSMC.

**Methods:**

Cultured aortic VSMC from SHR and WKY were used. Expression of 12-LO mRNA was determined by real-time polymerase chain reaction. Phosphorlyation of ERK1/2 and production of 12-LO and angiotensin II subtype 1 (AT_1_) receptor were assessed by Western blots. IL-8/CXCL8-stimulated DNA synthesis was determined by measuring incorporation of [^3^H]-thymidine. And effect of IL-8/CXCL8 on vascular tone was determined by phenylephrine-induced contraction of thoracic aortic rings.

**Results:**

Treatment with IL-8/CXCL8 greatly increased 12-LO mRNA expression and protein production compared to treatment with angiotensin II. IL-8/CXCL8 also increased the expression of the AT_1_ receptor. The increase in 12-LO induced by IL-8/CXCL8 was inhibited by treatment with an AT_1_ receptor antagonist. The induction of 12-LO mRNA production and the proliferation of SHR VSMC by IL-8/CXCL8 was mediated by the ERK pathway. The proliferation of SHR VSMC and the vascular contraction in the thoracic aortic ring, both of which were induced by IL-8/CXCL8, were inhibited by baicalein, a 12-LO inhibitor.

**Conclusion:**

These results suggest that the potential role of IL-8/CXCL8 in hypertensive processes is likely mediated through the 12-LO pathway.

## INTRODUCTION

Controlling chemokine production is important for regulating inflammatory reactions in hypertensive vascular walls. Inflammatory cell infiltration and oxidative stress in vascular walls contribute to the pathogenesis of hypertension, and the suppression of inflammatory cell infiltration has been shown to ameliorate hypertension in experimental animal models (1-5).

The chemokine IL-8/CXCL8 has been known to play an important role in monocyte migration into the subendothelial space in the early phase of atherosclerosis. In addition, elevated levels of IL-8/CXCL8 are associated with an increased risk of future coronary artery disease (6,7). We have previously demonstrated that the expression of IL-8/CXCL8 in aortic tissue and in vascular VSMC in SHR was higher than in VSMC from normotensive WKY (8).

IL-8/CXCL8 was shown to increase 12-lipoxygenase (12-LO) mRNA expression and protein production in porcine aortic VSMC (9). The 12-LO pathway of arachidonic acid metabolism has been linked to cell growth and to the pathology of hypertension (10-12). Angiotensin II (Ang II) is a potent positive regulator of 12-LO activation and expression in porcine and human VSMC (13,14). Increased levels of 12-LO induced by cytokines in porcine VSMC and an elevated level of 12-LO activity in SHR plasma have been reported (9,15). However, neither the mechanism of IL-8/CXCL8 induction of 12-LO expression nor the association between IL-8/CXCL8 and the 12-LO pathway specific to SHR VSMC have been studied. Therefore, we investigated the mechanism of action of IL-8/CXCL8 in relation to the expression of 12-LO in SHR VSMC.

## MATERIALS AND METHODS

### Reagents

The Trizol reagent for total RNA isolation was purchased from Invitrogen (Carlsbad, CA). PBS, DMEM, penicillin-streptomycin and FBS were purchased from Gibco/BRL (Life Technologies, Gaithersburg, MD). Recombinant human IL-8/CXCL8 was purchased from R&D systems (Minneapolis, MN). Baicalein was obtained from Sigma Chemical Co. (St Louis, MO). Ang II was obtained from Calbiochem (San Diego, CA). 12-Hydroxyeicosatetraenoic acid (12-HETE) was purchased from Cayman Chemical (Ann Arbor, MI). Losartan was obtained from MSD (Delaware, MD). MAPK inhibitor and 2'-amino-3' methoxyflavone (PD98059) were purchased from Calbiochem. Nitrocellulose transfer membranes were obtained from Schleicher & Schuell Bioscience (Dassel, Germany). Oligonucleotide primers for PCR of 12-LO, the AT_1_ receptor, the AT_2_ receptor and β-actin were synthesized by Bionics (Seoul, Korea). LightCycler FastStart DNA SYBR Green I Mix was obtained from Roche (Mannheim, Germany). The 12-LO antibody was purchased from Santa Cruz Biotechnology (Santa Cruz, CA). The AT_1_ receptor antibody was purchased from Abcam (Cambridge, UK). The phospho-ERK antibody was obtained from Cell Signaling Technology (Danvers). The g-tubulin antibody was obtained from Sigma Chemical Co. (St Louis, MO). All other reagents were from pure-grade commercial preparations.

### Experimental animals

Specific pathogen-free male inbred WKY and SHR, 20 to 30 weeks of age, were purchased from Japan SLC Inc. (Shizuoka, Japan). All experimental animals received autoclaved food and bedding to minimize exposure to viral and microbial pathogens. The rats were cared for in accordance with the Guide for the Care and Use of Experimental Animals of Yeungnam Medical Center.

### VSMC preparation

VSMC were obtained by an explant method from the thoracic aortas of 20- to 30-week-old male SHR and WKY as described by Kim et al. (8). VSMC were cultured in DMEM supplemented with 10% FBS and 1% penicillin-streptomycin. Cells were detached with 0.25% trypsin/EDTA and seeded into 75-cm^2^ tissue culture flasks at a density of 10^5^ cells per milliliter. All experiments were conducted during cell passages 3 to 7. Prior to stimulation, 95% confluent VSMC were serum-starved overnight by incubating in DMEM with 0.1% FBS. Cell cultures were incubated in a humidified incubator at 37℃ and 5% CO_2_ in the presence or absence of stimuli for the indicated times.

### Preparation of total RNA and real-time PCR

Total RNA was extracted using the Trizol reagent according to the manufacturer's instructions. The quantity of total RNA obtained was determined by measuring the optical density (OD) at 260 and 280 nm. Real-time PCR amplification of 12-LO, the AT_1_ receptor and the AT_2_ receptor from VSMC was performed using a LightCycler (Roche, Germany). cDNA was obtained by reverse transcription using 1µg of total RNA and then subjected to real-time PCR. PCR was performed in triplicate. Each 20-µl reaction contained LightCycler FastStart DNA SYBR Green I mix (Roche, Germany), primer and 2µl of cDNA. Prior to PCR amplification, the mixture was incubated at 95℃ for 10 min. The amplification consisted of 45 cycles of denaturation (10 s at 95℃), annealing (5 s at the primer-appropriate temperature), and extension (10 s at 72℃) with fluorescence detection at 72℃ after each cycle. After the final cycle, melting point analysis of each sample was performed over the range of 65 to 95℃ with continuous fluorescence detection. Expression levels of β-actin were used for sample normalization. The results for each gene are expressed as the expression level relative to the expression level of β-actin. The primers used for PCR were as follows: 12-LO (312 bp) sense, 5'-tggggcaactggaagg-3', antisense, 5'-agagcgcttcagcaccat-3'; AT_1_ receptor (445 bp) sense, 5'-cacctatgtaagatcgcttc-3', antisense, 5'-gcacaatcgccataattatcc-3'; AT_2_ receptor (65 bp) sense, 5'-ccgtgaccaagtcttgaagatg-3', antisense, 5'-agggaagccagcaaatgatg-3'; β-actin (101 bp) sense, 5'-tactgccctggctcctagca-3', antisense, 5'-tggacagtgaggccaggatag-3'. The levels of 12-LO, AT_1_ receptor and AT_2_ receptor mRNA were determined by comparing experimental levels to standard curves. mRNA levels were expressed as the fold increase or decrease of expression.

### Western blotting

Total lysates were prepared in PRO-PREP buffer (iNtRON, Seoul, Korea). Protein concentrations were determined by a Bradford assay (Bio-Rad, CA) using bovine serum albumin as a standard. Thirty micrograms of the protein samples were separated on 10% SDS-polyacrylamide gels and then transferred to nitrocellulose membranes. The membranes were soaked in 5% nonfat dried milk in TBST (10 mmol/L Tris-HCl pH 7.5, 150 mmol/L NaCl and 0.05% Tween-20) for 1 h and then incubated for 16~18 h at 4℃ with primary antibodies against 12-LO, the AT_1_ receptor, phospho-ERK and γ-tubulin. The membranes were washed three times with TBST for 10 min and then incubated with HRP-conjugated secondary antibody for 1 h at 4℃. The membranes were rinsed three times with TBST for 10 min, and antigen-antibody complexes were detected using an enhanced chemiluminescence detection system (LAS-3000; Fujifilm, Tokyo, Japan).

### VSMC proliferation

VSMC were plated in 24-well plates for 24 h and then exposed to the stimulant. [^3^H]-thymidine (1 µCi/ml) (Perkin-Elmer, Boston, MA) was added to the plates during the final 48 h of incubation. The cells were subsequently washed three times with cold PBS. [^3^H]-thymidine-labeled cells were collected with 0.1% SDS, and radioactivity was measured using a Packard scintillation counter (Packard instrument company, Meriden).

### Measurement of vascular tone

The thoracic aorta was cleared of adherent periadventitial fat and cut into rings 3~4 mm in width. To assess the direct effects of IL-8/CXCL8 on the vascular smooth muscle, the endothelium was removed by gently rubbing the lumenal surface with a needle. The rings were mounted in an organ bath filled with Krebs solution (pH 7.4: in mmol/L: 118 NaCl, 4.7 KCl, 2.5 CaCl_2_, 1.2 MgSO_4_, 1.2 KH_2_PO_4_, 5.7 glucose, 25 NaHCO_3_) at 37℃ and 95% O_2_-5% CO_2_. Isometric force was measured with a force transducer (FT03, Grass Instrument). A tension of 1 g was applied, and the rings were equilibrated for 60 min. During this process, the Krebs solution was changed every 15 min. The rings were then challenged with phenylephrine (PE, 10 µmol/L) to ensure tissue viability and with acetylcholine (Ach, 10 µmol/L) to ensure the absence of an endothelium. After inducing maximal contraction with the PE challenge, the vessels were incubated with IL-8/CXCL8 (200 ng/ml) and baicalein (10 µmol/L) for 1 h. After incubation with IL-8/CXCL8 and baicalein, a cumulative phenylephrine dose-response curve was constructed (1~100 µmol/L).

### Statistical analysis

The results are expressed as the mean±SD of at least three independent experiments. For comparisons among multiple groups, statistical significance was determined using the Mann-Whitney test using SPSS version 12.0.

## RESULTS

### Effect of IL-8/CXCL8 on 12-LO in SHR VSMC

**Figure 1:**
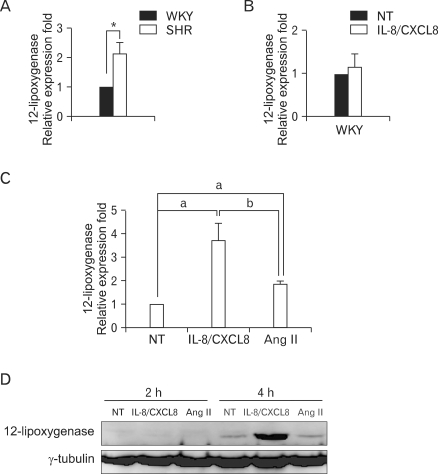
IL-8/CXCL8 increases 12-LO mRNA expression and protein production in SHR VSMC. (A) SHR and WKY VSMC were isolated from the thoracic aorta and cultured on plastic dishes at early passages (3 to 7). Total RNA was analyzed by real-time PCR. Bars represent means±SD from three independent experiments. ^*^p<0.05 vs. WKY VSMC. (B) WKY VSMC were untreated or treated with IL-8/CXCL8 (100 ng/ml) for 2 h. Total RNA was then analyzed by real-time PCR. Bars represent means±SD from three independent experiments. (C) SHR VSMC were untreated or treated with IL-8/CXCL8 (100 ng/ml) or Ang II (100 nmol/L) for 2 h. Total RNA was then analyzed by real-time PCR. Bars represent means±SD from three independent experiments. a: p<0.05 vs. untreated VSMC. b: p<0.05 vs. treated with IL-8/CXCL8. (D) SHR VSMC were untreated or treated with IL-8/CXCL8 (100 ng/ml) or Ang II (100 nmol/L) for 2 and 4 h. Cell lysates were separated on 10% SDS-polyacrylamide gels and then immunoblotted with the 12-LO antibody. Data shown are representative of three independent experiments.

We compared 12-LO expression in both SHR VSMC with the expression in and WKY VSMC. Expression of 12-LO mRNA was found to be higher in SHR VSMC relative to WKY VSMC (Fig. 1A). In addition, IL-8/CXCL8 treatment did not increase the expression level of 12-LO mRNA in WKY VSMC (Fig. 1B). We next examined the expression of 12-LO in SHR VSMC that had been treated with IL-8/CXCL8. In contrast, IL-8/CXCL8 treatment in SHR VSMC greatly induced the expression of 12-LO mRNA compared to Ang II treatment (Fig. 1C). Large amounts of 12-LO protein were detected in SHR VSMC that had been treated with IL-8/CXCL8 for 4 h (Fig. 1D).

### Effect of the AT_1_ receptor on IL-8/CXCL8-induced 12-LO expression in SHR VSMC

**Figure 2:**
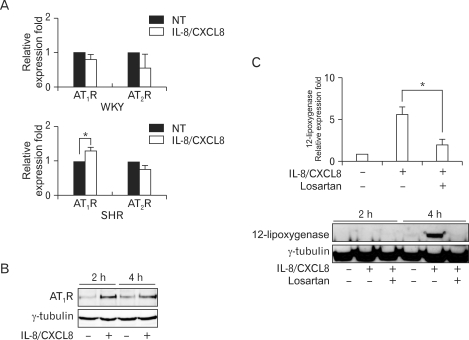
IL-8/CXCL8 increases AT_1_ receptor expression in SHR VSMC, and IL-8/CXCL8-induced expression of 12-LO mRNA is mediated through the AT_1_ receptor. (A) WKY and SHR VSMC were untreated or treated with IL-8/CXCL8 (100 ng/ml) for 2 h, and total RNA was analyzed by real-time PCR. Bars represent means±SD from four independent experiments. ^*^p<0.05 vs. untreated VSMC. (B) VSMC were untreated or treated with IL-8/CXCL8 (100 ng/ml) for 2 and 4 h. Cell lysates were separated on 10% SDS-polyacrylamide gels and then immunoblotted with the AT_1_ receptor antibody. Data shown are representative of three independent experiments. (C) VSMC were untreated or treated with IL-8/CXCL8 (100 ng/ml) or IL-8/CXCL8 plus losartan (AT_1_ receptor antagonist, 10µmol/L) for 2 h or 4 h, and the total RNA and cell lysates were isolated. Total RNA was analyzed by real-time PCR. Cell lysates were separated on 10% SDS-polyacrylamide gels and then immunoblotted with the 12-LO antibody. Bars represent means±SD from three independent experiments. ^*^p<0.05 vs. VSMC treated with IL-8/CXCL8. Data shown are representative of three independent experiments.

To determine whether IL-8/CXCL8-induced 12-LO production is mediated by the AT_1_ receptor, we first examined the effect of IL-8/CXCL8 on AT_1_ and AT_2_ receptor mRNA expression in WKY and SHR VSMC. IL-8/CXCL8 did not increase the expression of AT_1_ or AT_2_ receptor mRNA in WKY VSMC (Fig. 2A). However, treatment with IL-8/CXCL8 increased the expression of AT_1_ receptor mRNA in SHR VSMC (Fig. 2A). AT_1_ receptor protein production was also detected in SHR VSMC (Fig. 2B). The AT_1_ receptor antagonist losartan (10 µmol/L) was found to significantly inhibit IL-8/CXCL8-induced expression of 12-LO mRNA. The production of the 12-LO protein was also inhibited by this antagonist (Fig. 2C). These results suggest that the induction of 12-LO expression by IL-8/CXCL8 is mediated through the AT_1_ receptor expression in SHR VSMC.

### IL-8/CXCL8-induced 12-LO expression is mediated by ERK phosphorylation

**Figure 3:**
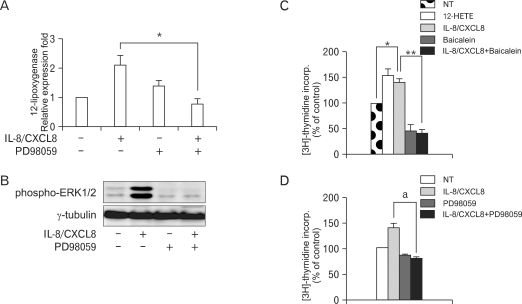
Expression of IL-8/CXCL8-induced 12-LO is mediated through the ERK pathway, and proliferation of SHR VSMC by IL-8/CXCL8 is inhibited by baicalein and PD98059. (A, B) VSMC were untreated (NT) or pretreated with PD98059 (ERK inhibitor, 10 µM) for 30 min. Cells were left untreated or treated with IL-8/CXCL8 (100 ng/ml) for 2 h, and the total RNA and cell lysates were isolated. The total RNA was analyzed by real-time PCR (A), and cell lysates were separated on 10% SDS-polyacrylamide gels and then immunoblotted with the phospho-ERK1/2 antibody (B). Bars represent means±SD from three independent experiments. ^*^p<0.05 vs. VSMC treated with IL-8/CXCL8. Data shown are representative of three independent experiments. (C, D) SHR VSMC were treated with 12-HETE (500 nmol/L), with IL-8/CXCL8 (100 ng/ml), with baicalein (12-LO inhibitor, 10 µmol/L, B), or with PD98059 (10 µmol/L, C) for 48 h in medium containing [^3^H]-thymidine (1 µCi/ml). [^3^H]-thymidine incorporation is shown on the Y-axis. Bars represent means±SD from three independent experiments run in triplicate. ^*^p<0.05 vs. untreated VSMC. ^**^p<0.01 vs. VSMC treated with IL-8/CXCL8 alone. a: p<0.05 vs. VSMC treated with IL-8/CXCL8 alone.

We investigated whether the MAPK signaling pathway is involved in IL-8/CXCL8-induced 12-LO expression. The IL-8/CXCL8-induced expression of 12-LO mRNA was decreased by PD98059 (Fig. 3A), and a high level of phosphorylation of ERK in SHR VSMC treated with IL-8/CXCL8 was detected (Fig. 3B) 

### Effect of 12-LO and ERK activation on IL-8/CXCL8-induced VSMC proliferation

It has been reported that IL-8/CXCL8 induces rat and human VSMC proliferation (16). Having established that IL-8/CXCL8 enhances 12-LO expression and stimulates VSMC proliferation, we evaluated the effect of 12-HETE, the product of 12-LO catalysis, on SHR VSMC proliferation. Fig. 3C shows that 12-HETE directly stimulates VSMC proliferation. In addition, baicalein, which is an inhibitor of 12-LO, significantly inhibited the IL-8/CXCL8-induced proliferation of SHR VSMC. IL-8/CXCL8-induced SHR VSMC proliferation was also completely blocked by PD98059 (Fig. 3D). These results suggest that ERK and 12-LO are linked in the IL-8/CXCL8-induced SHR VSMC proliferation pathway.

### Effect of 12-LO on IL-8/CXCL8-induced vascular tone contraction

**Figure 4:**
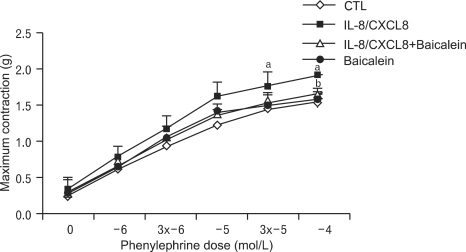
Effect of IL-8/CXCL8 on the phenylephrine-induced contraction of thoracic aortic rings. Phenylephrine (1~100 µmol/L) was added to isolated thoracic aortic rings pretreated with IL-8/CXCL8 (200 ng/mL) and/or the 12-LO inhibitor baicalein (10 µmol/L) for 1 h. All contractions are expressed as grams (g) of contractile tension in control rings not exposed to IL-8/CXCL8 or baicalein. Data are means±SD of three independent experiments. a: p<0.05 vs. control rings. b: p<0.05 vs. rings treated with IL-8/CXCL8 alone.

We also examined the potential role of 12-LO in mediating the vascular contraction effects of IL-8/CXCL8. This was done by assessing the effect of 10 µmol/L baicalein on IL-8/CXCL8-induced vascular contractions. The endothelium of the aortic rings was removed to determine the direct effect of IL-8/CXCL8 on vascular smooth muscle. The force of vascular contractions induced by IL-8/CXCL8 was generated greater than those of the control group. However, baicalein considerably inhibited the contraction-inducing effect of IL-8/CXCL8 (Fig. 4).

## DISCUSSION

AT_1_ receptor mediates most of pathophysiological actions of Ang II. Whereas stimulation of the AT_1_ receptor leads to activate cytokines and adhesion molecules expression, cell growth, angiogenesis and vasoconstriction, AT_2_ receptor stimulation causes opposite effects, including apoptosis, anti-angiogenesis, and vasodilatation (17-19). AT_1_ receptor is widely expressed in all organs and, especially, VSMC expresses AT_1_ receptors at ordinary times (20,21). In our study, we detected the expression of AT_1_ receptor mRNA in VSMC both of from SHR and WKY, however, the protein production of AT_1_ receptor was very weak in SHR VSMC (Fig. 1). IL-8/CXCL-8 increased AT_1_ receptor mRNA expression in SHR VSMC only, and confirmed strong production of AT_1_ receptor protein (Fig. 2B). Therefore, we observed the effects of AT_1_ receptor antagonists on IL-8/CXCL8-induced 12-LO production in SHR VSMC. AT_1_ receptor antagonist significantly inhibited IL-8/CXCL8-induced 12-LO production (Fig. 2C). These results suggest that the action of IL-8/CXCL8 on hypertensive vasculature is likely to be associated with the AT_1_ receptor.

ERK pathway was involved in IL-8/CXCL8-induced 12-LO expression in SHR VSMC. The IL-8/CXCL8-induced expression of 12-LO mRNA was decreased remarkably by PD98059, an inhibitor of ERK (Fig. 3A). We also examined whether p38 signaling pathway is involved in IL-8/CXCL8-induced 12-LO expression in SHR VSMC. PD169316, an inhibitor of p38, also decreased the expression of IL-8/CXCL8-induced 12-LO mRNA in SHR VSMC. However, it did not inhibit significantly IL-8/CXCL8-induced 12-LO mRNA expression compared to PD98059 (data not shown).

IL-8/CXCL8 has been shown to have mitogenic effects on VSMC (16), and Natarajan et al. (9) reported that 12-LO inhibition decreased IL-8/CXCL8-induced proliferation in porcine VSMC. We also found that baicalein inhibited IL-8/CXCL8-induced proliferation of SHR VSMC and that SHR VSMC proliferation induced by IL-8/CXCL8 was greater than Ang II-induced proliferation (data not shown). ERK activation has been demonstrated in the normal growth of mesangial cells and pulmonary artery smooth muscle cells in association with the stimulation of the 12-LO pathway (10,22). In this study, IL-8/CXCL8-induced 12-LO expression was mediated through ERK activation. In addition, IL-8/CXCL8-induced VSMC proliferation was completely blocked by PD98059. These results suggest that ERK activation and 12-LO expression are linked in IL-8/CXCL8-induced VSMC proliferation and that 12-LO induction precedes ERK activation.

Buemi et al. (23) suggested that IL-8/CXCL8 may directly enhance membrane permeability to Ca^2+^, thus inducing vasoconstriction in the smooth muscle cells of patients with essential hypertension. However, Ohkawa et al. (24) reported that IL-8/CXCL8 had no significant effect on the vascular contraction of the thoracic aorta in Sprague-Dawley rats. In the present study, we observed a significant increase in the contraction of the thoracic aortic rings from SHR that were treated with IL-8/CXCL8, and this contraction decreased after baicalein treatment (Fig. 4). This result suggests that IL-8/CXCL8 acts on vascular contraction through the 12-LO pathway in SHR.

This study demonstrates that IL-8/CXCL8-induced 12-LO expression is mediated through the AT_1_ receptor in SHR VSMC and that the 12-LO pathway participates in IL-8/CXCL8-induced SHR VSMC proliferation, most likely via the ERK pathway. Taken together, our results suggest that the effect of IL-8/CXCL8 on hypertensive vascular walls is mediated by the induction of the 12-LO pathway.
